# Blastoid mantle cell lymphoma: cutaneous infiltration^☆^^☆☆^

**DOI:** 10.1016/j.abd.2020.06.025

**Published:** 2021-05-18

**Authors:** Mariana Righetto de Ré, Flávia de Oliveira Valentim, Mariangela Esther Alencar Marques, Silvio Alencar Marques

**Affiliations:** Department of Dermatology, Faculty of Medicine, Universidade Estadual Paulista, São Paulo, SP, Brazil

**Keywords:** B-lymphocytes, Lymphoma, Pathology, Skin neoplasms

## Abstract

Mantle cell lymphoma is an aggressive B-cell, non-Hodgkin’s lymphoma, with lymph node or extranodal origin, and a mean survival of three to five years. Skin involvement is rare, secondary and indicates neoplasia dissemination. The authors report a case of a female patient, 69 years old, diagnosed previously, after lymph node and bone marrow biopsy. She was undergoing the second chemotherapy regimen when she showed infiltrated plaque-like lesions, nodules and tumors on the trunk and thigh root. Histopathology and immunohistochemistry demonstrated cutaneous infiltration of the blastoid lymphoma.

## Introduction

Mantle Cell Lymphoma (MCL) is a B-cell, non-Hodgkin's lymphoma, derived from inner cells in the mantle zone of lymphoid follicles. It is an aggressive tumor, with a mean survival of three to five years.^1^ It corresponds to 4% of lymphomas diagnosed in the United States of America and between 7% and 9% in Europe, with a higher incidence in male patients and those over 60 years of age (2: 1).^1^, ^2^ It has four histological variants: small cells, from the mantle zone, diffuse and blastic variants, or blastoid, with the latter corresponding to between 10% and 30% of cases of MCL.^2^, ^3^ It occurs primarily in lymph nodes and extranodal organs, such as bone marrow and spleen. Cutaneous involvement is rare, secondary, and occurs in approximately 2% of cases, and can be understood as a sentinel for the lymphoma dissemination and more frequently associated with cytological characteristics of blastoid origin, also pointed out as having the worst prognosis.^4^ In the few cases of cutaneous involvement described in the literature, the most prevalent lesions consisted mainly of subcutaneous infiltrations, nodules and tumors.^4^

## Case report

The authors report a female patient, 69 years old, diagnosed previously with cutaneous mantle cell lymphoma through lymph node and bone marrow biopsy, after complaining of generalized painful lymph node enlargement, associated with weight loss, asthenia and gastrointestinal pain.

**Figure 1:**
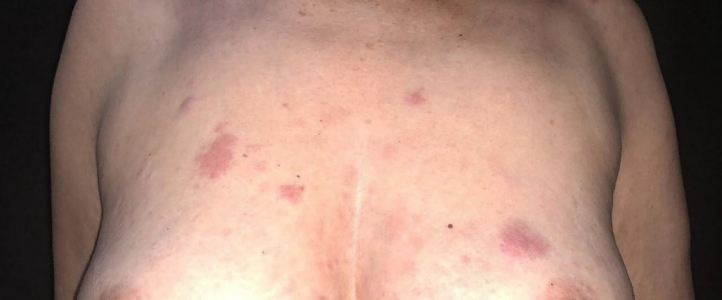
Cutaneous mantle-cell lymphoma. Infiltrated purplish erythematous papules and plaques, located on the upper thorax.

**Figure 2:**
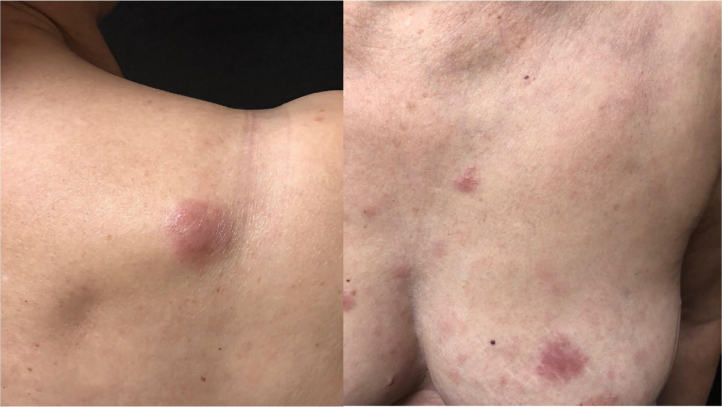
Infiltrated plaque in the scapular region and infiltrated papules and plaque on the breast and sternal regions.

**Figure 3:**
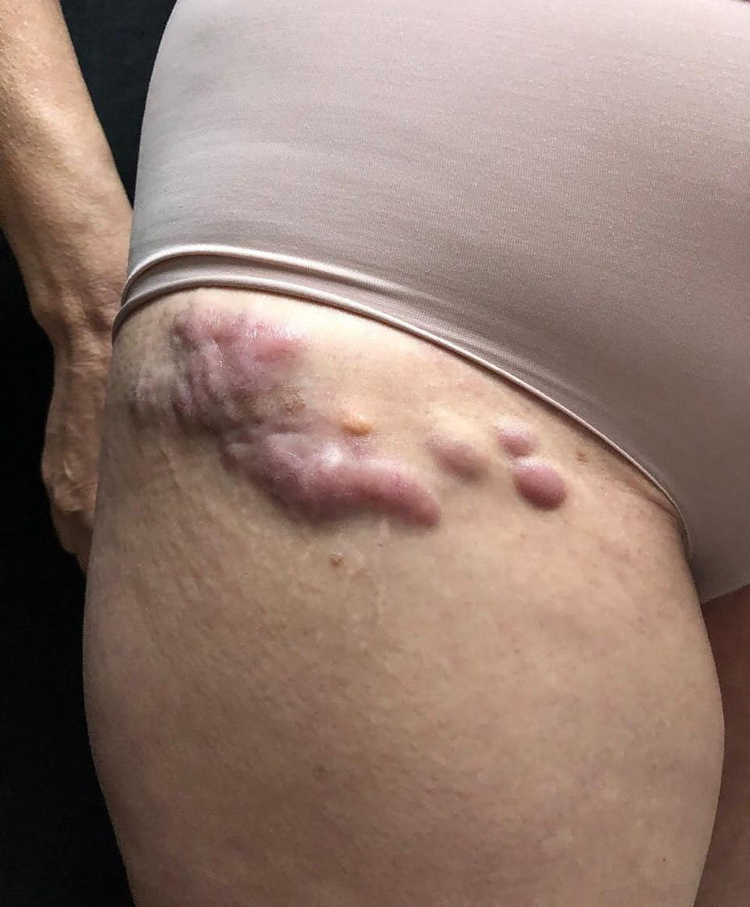
Cutaneous mantle-cell lymphoma. Erythematous-brownish nodules and tumors on the right inguinal crural region.

She was initially treated with the CHOP regimen, alternating with HD-araC, with a full response after the seventh cycle. However, disease recurrence was diagnosed in the seventh month after the diagnosis and she has transitioned to the GEMOX-FT (gemcitabine and oxaliplatin) regimen. However, a few months later, while undergoing this treatment regimen, she successively presented, erythematous lesions, papules, nodules, and infiltrated plaques on the trunk and abdomen (Figure 1, Figure 2). Subsequently, tumor/nodular lesions appeared in the inguinal region (Fig. 3), associated with recurrence of diffuse lymph node enlargement, occasion when the Dermatology service was consulted.

**Figure 4:**
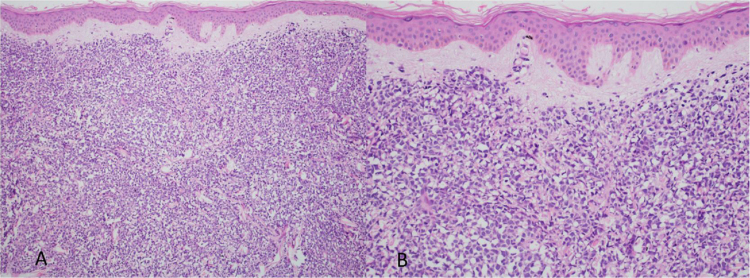
Cutaneous mantle-cell lymphoma. (A) Tumor infiltration in the dermis by small and medium-sized lymphocytes (Hematoxylin & eosin, ×40). (B) Higher magnification showing the Grenz zone isolating the infiltrate from the epidermis (Hematoxylin & eosin, ×100).

**Figure 5:**
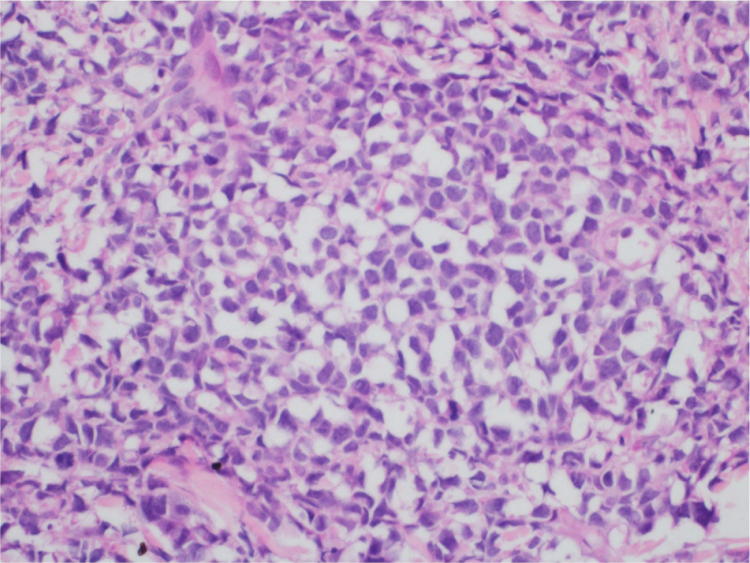
Detail of the neoplastic infiltration with an accumulation of blastoid cells, with large, irregular nuclei and scarce cytoplasm. (Hematoxylin & eosin, ×200).

**Figure 6:**
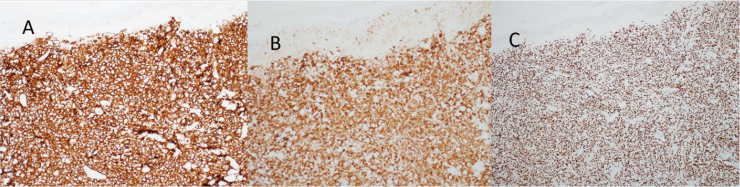
Cutaneous mantle-cell lymphoma. Immunohistochemistry staining: (A) CD20, (B) Cyclin-D1 and (C) PAX5.

At the examination, the infiltrated plaques were erythematous, firm, and the tumor/nodule lesions were erythematous-brown, firm and painless. The clinical hypothesis was specific skin infiltration due to the underlying disease, confirmed after biopsy of an infiltrated plaque. The corresponding anatomopathological examination showed dense skin infiltration of the dermis and subcutaneous cellular tissue by small and medium-size lymphocytes, with irregular nuclei and accumulation of blast cells. The infiltrate spared the epidermis and maintained the Grenz zone visible, occupying the superficial and papillary dermis (Figure 4, Figure 5). Immunohistochemical analysis revealed positive staining for CD20, CD5, PAX5, and Cyclin D1, as well as negative staining for CD23 and CD30, diagnostic findings of blastoid mantle cell lymphoma with cutaneous infiltration (Fig. 6). After histopathological confirmation, the chemotherapy regimen was modified again to cycles of ifosfamide, etoposide and dexamethasone, with initial remission of the disease. However, approximately two years after the diagnosis, the patient died due to the systemic dissemination of the lymphoma.

## Discussion

Mantle cell lymphoma is associated with a mutation characterized by the chromosomal translocation t(11:14) (q13 32) of the CCND1 gene, leading to the aberrant expression of Cyclin D1, which is not typically expressed in normal lymphocytes.^2^, ^3^ This increased expression of the Cyclin D1 protein leads to the dysregulation of the normal cell cycle, as it acts as a positive signal for the transition from G1 to S phase of cell division and stimulates uncontrolled cell proliferation.

Immunophenotypically, it consists of tumor cells positively stained for CD20+, CD5 +, cyclin-D1+, but negative for CD3, CD10, CD23, CD30, and bcl-6.^2^, ^3^ In the anatomopathological study, the typical morphology is that of monomorphic lymphoid cells, small to medium in size, with irregular nuclear contours, and the accumulation of large cells, with large nuclei and scarce cytoplasm in the blastoid variant.^4^, ^5^

Clinically, it is initially expressed as B symptoms, that is generalized lymph node enlargement, bone marrow infiltration and splenomegaly; this last one may have an important impact.^1^, ^2^ Other affected organs are the gastrointestinal tract and, more rarely, ocular, cutaneous integument, and CNS infiltration. Regarding the laboratory tests, it is characterized by the presence of pancytopenia or a leukemic presentation with extensive leukocytosis.^1^, ^2^ The blastoid variety of MCL occurs in 10% to 30% and is associated with worse prognosis.^2^

Due to the low frequency of MCL with cutaneous involvement, few cases have been described in the literature, and until 2015, only 25 cases were described in reports written in the English language. Based on these data, the most prevalent lesions are nodule-like, but macules, infiltrated plaques, tumors, and subcutaneous infiltrates may also be present.^4^, ^5^, ^6^ The most frequently affected locations are the trunk and face, followed by the limbs and scalp. It should be noted that the presence of cutaneous lesions signals worse prognosis and may be a warning sign of the presence of systemic MCL.^7^ Primary mantle cell lymphoma of the skin is extremely rare and requires extensive and detailed systemic investigation to exclude extracutaneous involvement, for it to be considered as such.^8^

Regarding therapy, due to the rarity of MCL, there are no randomized clinical trials that reported which treatment is the most appropriate and, in clinical practice, the CHOP regimen or its variations are used as the initial treatment.^9^, ^10^ With the use of these treatment initiatives, remissions are observed in the short term; however, as in the present case, they are followed by recurrence and fatal evolution. Rituximab has been proposed as an alternative monotherapy or associated with different regimens. Moreover, bone marrow transplantation should also be considered.^9^, ^10^

The present report shows the dermatological and histopathological manifestations of mantle cell B-lymphoma, of the blastoid variety, a rare, aggressive lymphoma with multiple skin lesions indicative of dissemination and poor prognosis.

## Financial support

None declared.

## Authors’ contributions

Mariana Righetto de Ré: approval of the final version of the manuscript; design and planning of the study; drafting and editing of the manuscript; critical review of the literature.

Flávia de Oliveira Valentim: approval of the final version of the manuscript; critical review of the manuscript.

Mariangela Esther Alencar Marques: approval of the final version of the manuscript; collection, analysis, and interpretation of data; critical review of the manuscript.

Silvio Alencar Marques approval of the final version of the manuscript; drafting and editing of the manuscript; critical review of the literature; critical review of the manuscript.

## Conflicts of interest

None declared.
